# Public Attention and Sentiment toward Intimate Partner Violence Based on Weibo in China: A Text Mining Approach

**DOI:** 10.3390/healthcare10020198

**Published:** 2022-01-20

**Authors:** Heng Xu, Jun Zeng, Zhaodan Tai, Huihui Hao

**Affiliations:** 1 School of Management, Henan University of Technology, Zhengzhou 450001, China; junzeng@stu.haut.edu.cn; 2 School of Foreign Languages, Henan University of Technology, Zhengzhou 450001, China; taizhaodan@haut.edu.cn; 3 Faculty of Agribusiness and Commerce, Lincoln University, Lincoln 7647, New Zealand; huihui.hao@lincolnuni.ac.nz

**Keywords:** intimate partner violence (IPV), social network site (SNS), public attention, text mining, sentiment analysis

## Abstract

The mobile internet has resulted in intimate partner violence (IPV) events not being viewed as interpersonal and private issues. Such events become public events in the social network environment. IPV has become a public health issue of widespread concern. It is a challenge to obtain systematic and detailed data using questionnaires and interviews in traditional Chinese culture, because of face-saving and the victim’s shame factors. However, online comments about specific IPV events on social media provide rich data in understanding the public’s attitudes and emotions towards IPV. By applying text mining and sentiment analysis to the field of IPV, this study involved construction of a Chinese IPV sentiment dictionary and a complete research framework. We analyzed the trends of the Chinese public’s emotional evolution concerning IPV events from the perspectives of a time series as well as geographic space and social media. The results show that the anonymity of social networks and the guiding role of opinion leaders result in traditional cultural factors such as face-saving and family shame for IPV events being no longer applicable, leading to the spiral of an anti-silence effect. Meanwhile, in the process of public emotional communication, anger often overwhelms reason, and the spiral of silence remains in effect in social media. In addition, there are offensive words used in the IPV event texts that indicate misogyny in emotional, sexual, economic and psychological abuse. Fortunately, mainstream media, as crucial opinion leaders in the social network, can have a positive role in guiding public opinion, improving people’s ability to judge the validity of network information, and formulating people’s rational behaviour.

## 1. Introduction

Intimate partner violence (IPV) is the most common type of domestic violence and a major public health problem [1]. About 35% of the world’s population has experienced intimate partner violence or non-partner sexual violence in their lifetime [2]. *China Central Television* (*CCTV*) reported that about 20% of households experience domestic violence in China. Since the COVID-19 outbreak, intimate partner violence (IPV) reports have increased globally. Mainland China’s first law to prevent domestic violence, the “Antidomestic Violence Law of the People’s Republic of China”, was formally implemented on 1 March 2016. The law clearly defines domestic violence as “the use of beatings, restrictions, and intentional disability, restrictions on personal freedom and frequent verbal abuse or intimidation characterized by physical, psychological or any other illegal acts among family members” [3]. With antidomestic violence legislation and implementation, domestic violence issues have gradually entered the public eye and aroused widespread public concern.

In the Chinese community, IPV is regarded as a private matter [4]. People often have the notion that family ugliness should not be publicized, and wish to keep family affairs private to reduce their sense of shame [5]. Traditional Eastern culture, including concerns about embarrassment, concern for image, and the shame of victims, results in IPV being underreported [6], limiting our understanding of its nature. However, due to the spread of the mobile internet, IPV events involving celebrities have gradually been exposed to the public eye [7].

In the afternoon of 25 November 2019, the International Antidomestic Violence Day, a beauty blogger (known as *Yuya* by Chinese netizens, female, 28 years old) released a 12-min video on her personal Sina Weibo account (*@YUYAMIKA*) to disclose the fact of her suffering domestic violence inflicted by her ex-boyfriend (Weibo account: *@Tuotuodefengmojiao*, known as *Tuotuo* by Chinese netizens).

Due to the huge number of page views and heated discussions because of the celebrity effect, the IPV incident “*Yuya* suffering from domestic violence” (hereafter called *Yuya*) immediately topped the hot topics of Sina Weibo and sparked a public discussion. As of 17 February 2020, the topic of “*Yuya* suffering from domestic violence” on Weibo had been clicked and viewed 3.16 billion times and discussed with 333,000 comments. The forwarding volume of the four microblogs released by *Yuya* reached 472,000, and the number of likes reached 5,217,000. Moreover, a lot of user-generated content about the IPV event was generated in China’s social networks. For example, “*Yuya* does not know that so many women are suffering from domestic violence”, “*Yuya*’s ex-boyfriend is detained”, “Chongqing (a municipality of China) Women’s Federation intervenes in domestic violence suffered by *Yuya*” and “*China Central Television (CCTV)* interviews with *Yuya*”. During the same period, 63 related microblog topics broke out, the number of views reached 4.29 billion and the number of discussions reached 412,000.

The *Yuya* event is not an independent case, and it concludes a series of events related to *Yuya* in the social network. It has become the only IPV event in the past ten years that has caused great repercussions nationwide in the development of China’s social media. The sudden IPV event resulted in the public actively seeking information and sharing opinions on social media [1], such as Weibo. As the most widely used social media platform in China [8], Weibo has been used in scientific research [9]. Therefore, the comment texts on social media related to this specific event can provide rich data for understanding the public’s emotional orientation and focus on IPV [7]. In this context, our research used text mining and sentiment analysis methods to study the public’s dynamic attention and sentiment evolution towards IPV, and proposes suggestions for increasing IPV focus in China.

The content of this research and the rest of the paper is as follows. Section 2 reviews the literature on public concern, emotion, and acceptance of IPV. Section 3 introduces the methodology and constructs a sentiment analysis framework including three steps: (1) data preprocessing; (2) constructing a sentiment dictionary related to IPV, and (3) sentiment analysis. Taking *Yuya* as an example, Section 4 uses the text of Weibo users’ comments to analyze the public’s attention and sentiment evolution towards IPV, analyzes the character and changing trends of users’ attention on IPV and analyzes the characteristics of the period, and regional characteristics. Section 5 further discusses the misogyny characteristics of public opinion and the breaking of the spiral of silence effect by the public under the background of IPV. Section 6 discusses the Delphi method used to prove our findings. Section 7 draws the conclusions.

## 2. Literature Review

IPV often replaces concepts such as violence against women (VAW) and domestic violence (DV) [10]. In most studies [11,12,13,14,15,16], IPV is defined as psychological, physical, and sexual violence, and some studies [17,18] involve economic violence. Economic IPV refers to controlling the resources of a current or former partners’ resources, trying to limit their economic activities, driving people out of their homes, or not providing financial support to families when resources are available [19]. In particular, cold violence often occurs in intimate partner relationships in China, but people often do not regard this behavior itself as IPV [20]. In addition, the “China Anti-Domestic Violence Law” explicitly prohibits manifestation of IPV between spouses, and clearly states that violence against other family members, such as the elderly and children, is also applicable to this law.

IPV is a significant public health issue affecting human beings. IPV has received widespread attention in the West, especially in the United States [13,16,21,22,23]. These studies found that different types of IPV are related to age, relationship status, family income, residential area, and childhood violence [24]. Although IPV is a term with obvious gender neutrality, women are the most common victims, and the perpetrators are primarily men [4]. Based on data from the third China Women’s survey (SSCW3) in 2010, the study of Breckenridge et al. [25] showed the gender differences and reported the lifetime prevalence of marital victims: psychological violence (female: 24.9%, male: 22.8%), physical violence (female: 5.5%, male: 2.5%) and sexual violence (female: 1.7%, male: 0.3%). Based on relevant data from the Demographic and Health Survey (DHS) of 23 countries, Aboagye et al. [26] found that women who support IPV are more likely to experience IPV than women who reject IPV. However, based on the interview data of 3740 Hong Kong couples, and comparing the self-reports between partners, Chan [4] concluded that there is gender symmetry in the prevalence of IPV; that is, women are as violent as men, and most IPV behaviors are mutual and two-way.

In contrast, key information on IPV in China is still lacking [15]. IPV has become an integral part of nursing research in Hong Kong and Taiwan, and different methods have been used to study various aspects of IPV [27]. However, in mainland China and Macau, IPV has always been an overlooked issue in nursing, and IPV research is limited in quantity, quality, and diversity [27]. The research content of Chinese scholars mainly focuses on the popularity and characteristics, risks and protective factors, legal responses, attitudes, and beliefs related to IPV [28,29,30]. With the promulgation of the “China Anti-Domestic Violence Law”, IPV has also received increasing attention from academic circles in mainland China, such as the impact of COVID-19 on domestic violence in China [1], police response to IPV [31,32], and a study of college students’ attitudes towards intimate partner violence [33].

### 2.1. Public Attitudes towards IPV

Some traditional attitudes in Chinese culture, such as “beating and scolding is a symbol of love”, and “IPV is a domestic shame and should not be made public” [30,34], may cause people to acquiesce or tolerate violence [35]. In particular, the traditional mindset of males being superior and females inferior still exists in some economically undeveloped areas and constrains many women. With their lower education level and economic independence, many victims have no idea of their legal rights and the methods to protect their rights. This is common in rural households with a higher incidence of domestic violence [36,37]. As a result, victims do not seek legal help in the first place but choose informal help, including extended family members, friends, and community members, to deal with violence [30,38]. However, as survivors relationships decline, they are more likely to seek support from a broader range of sources [30].

In China, the police usually identify IPV events, including most domestic violence cases, as ordinary family disputes without, or with little, criminal justice intervention [31]. Mediation, especially oral mediation by police officers, is still the most used method of handling of these kinds of events [39,40]. Therefore, cultural customs and traditional values affect the accurate analysis of IPV prevalence in China.

People’s perception of IPV relates to their gender [33]. Lin et al. [33] clearly stated that females are more likely to define specific behaviors as IPV than males, and those who show more positive attitudes towards male domination are relatively tolerant of IPV. Fanslow et al. [41]. Shen et al. [42] also found gender differences in perception of IPV.

In addition, socioeconomic status is a related factor that affects people’s view of IPV. People with higher socioeconomic status are more likely to define certain behaviors as IPV [33]. In real life, the occurrence of celebrity domestic violence incidents has received more attention and wider discussion. However, serious gender issues can easily lead to entertainment trends due to the celebrities’ social identities. Media reports may treat internet celebrities’ IPV events as entertainment, which affects the public’s attitude towards IPV events.

In fact, the media influences the public’s perception of IPV, either from a public health perspective or as a private issue in interpersonal relationships. Social media breaks the “gateway” barriers in traditional mass media, allowing the public to produce media content directly. Social networking platforms allow survivors to share their stories, promote these on behalf of survivors, and spread protection awareness [43]. Carlyle et al. [43] investigated how IPV information on Instagram reflects the public’s understanding of protection methods. Of the posts, 94% attributed IPV to the abuser, but only 21% attributed the responsibility for preventing IPV to the abuser. However, 43% of posts blamed the victim because the victim did not leave the abusive relationship.

When discussing IPV events, people tend to focus on the role of the victim. This view recognizes that such abuse on the victim may cause serious and lasting effects [43]. The media also has the ability to influence the public’s perception of IPV. Therefore, the public’s attitude towards IPV in social media is particularly important for preventing IPV.

### 2.2. Research Methods of IPV

The methods of IPV research are primarily quantitative, based on questionnaires and interviews involving many fields such as medicine, sociology, and psychology. Jewkes et al. [44] studied men and violence in Asia and the Pacific based on interviews using logistic regression and structural models. Žukauskienė et al. [24] investigated the prevalence of psychological, economic, physical violence, and IPV, the main modes of IPV exposure, and the interconnection between IPV and socio-demographic characteristics, by collecting data through face-to-face interviews. In addition, a comparative study of IPV perspectives between regions has also received attention. Based on the research samples of Chinese and American college students, Pugh et al. [45] investigated the causes of women’s abusive relationships, their attitudes towards IPV [33], and their response to IPV [46]. However, regional questionnaires have certain limitations, and the quality of their samples affected the accuracy of the survey.

The methods of the previous literature focused on questionnaires and interviews to obtain small-scale data (see Table 1), which is conducive to the study of a small number of groups; for example, the LGBT group. Existing IPV research is limited by observation data availability and lacks systematic analysis from the public perspective.

In traditional Chinese family culture, due to the face-saving factor, avoiding shame can potentially affect the behavior of respondents [25]. Compared with face-to-face interviews, questionnaire surveys have a certain degree of anonymity and are widely used in the field of IPV [15,16,22]. Inspired by the ecological model, Wei et al. [47] used anonymous online questionnaires to obtain survey data from 15 cities. They used univariate and multivariate regression analyses to explore the factors that cause IPV between homosexuals.

The data basis of IPV research in China can be divided into general population research and specific populations research based on national data. A survey on the status of Chinese women was jointly launched by the China Women’s Federation and the China Statistical Bureau, including individual questionnaires and community (village, neighborhood committee) questionnaires. The Third Survey on the Status of Chinese Women (SSCW3) defines IPV behavior and explains different types of IPV. In order to deeply analyze the social status and its changes of different female groups, this survey also investigated five typical groups, namely, children, the elderly, college students, people affected by migration and high-level talents. Most studies are based on the data of SSCW3 due to its authority and reliability, but SSCW3 ignores IPV data of a marginalized group, namely LGBT [50]. The research methods of questionnaires and interviews have regional limitations since the data can only be collected from a specific region. It is difficult to understand the views of the entire country or region of IPV from one data set.

The emergence of social media broadens the narrative surrounding the form of IPV to be more inclusive of sexual, economic, and psychological abuse, and the power of social media promotes IPV as a public health issue [51]. Although people are paying more and more attention to the potential of social media in social science research, the application of social media in the field of IPV research is limited [48]. Social media provides a platform for capturing the life experiences of IPV victims, and the development of social media helps to have a broader understanding of IPV. Storer et al. [48] used the data generated by the #WhyIStayed Twitter campaign to investigate why Twitter users maintained abusive relationships and found that the social manifestations of domestic violence have become obstacles for victims to leave the abusive relationship in various ways. This research shows that Twitter information can serve as a micro-narrative narrating the life experience of IPV victims [48], especially for hard-to-reach people during a special period [52]. Alvarez-Hernandez et al. [49] investigated the spread of IPV help messages by Spanish-language media in the United States during the COVID-19 pandemic and analyzed the data using qualitative methods to support the Latino community seeking help to solve the IPV problems. Karami et al. [53] offered a new approach to identify health-related tweets and topics. Therefore, future research should specifically explore social media data to investigate IPV survivors and collect more valuable and critical background information.

With the development of the Internet of Things and cloud computing technologies, data has exploded, giving rise to a fourth paradigm of scientific research in the context of big data; text mining is an indispensable part of this [54]. Text mining is a technology that processes massive text data (such as social media platforms) and extracts valuable information. By mining comment text, it is possible to understand the public’s attention to IPV events and trends. Digital technology, or big data, has the potential to become a rich source of data to understand social movements, social interactions and behaviors among users in virtual platforms and to investigate social phenomena [48].

In the context of big data, some scholars have used text data generated by social media to analyze the field of IPV. These studies obtained new academic results and proved that social media data are effective, can be a supplement to the data from surveys, and provide a broader perspective for the research in the field of IPV. Furthermore, the content and online comments on IPV events in social networks generated by a large number of users provide favorable conditions to understand the public’s attitude deeply.

Using the method of sentiment analysis technology, also known as opinion mining, people’s opinions, affections, evaluations, attitudes and emotions about entities and their attributes from social media posts can be analyzed to understand the public’s attitude, worry and panic about an IPV incident [55]. When using the method of sentiment analysis, many researchers used different analysis methods. Zhang et al. [56], Shakil et al. [57] and Phu et al. [58] used dictionary-based methods; Truică et al. [59], Messina et al. [60], Wang et al. [61] and other studies used machine learning, whereas Szabóová et al. [62], Ahmed et al. [63] combine the dictionary-based methods and machine learning. The sentiment dictionary-based method is good at processing fine-grained text sentiment analysis, which is conducive to analyzing sentiment characteristics in specific fields [64].

## 3. Methodology

**Figure 1:**
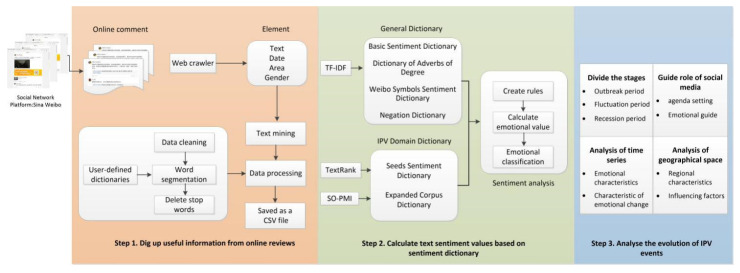
IPV sentiment analysis framework.

Based on the previous literature, this study updates a dictionary of adverbs of different degrees and expression sentiments. Then this study constructs an emotion dictionary suitable for the IPV field to explore the Chinese public’s attitude towards IPV using social media data. The research framework includes three stages, as shown in Figure 1.

Step 1: Select microblog comments related to IPV events and use Python to conduct data crawling and complete the preprocessing of text data.

Step 2: Build an IPV sentiment dictionary and establish the calculation rules to calculate the sentiment value of text sentences.

Step 3: Divide the development stages of IPV events and analyze each stage’s public attitude and emotional features.

### 3.1. Mining Useful Information from Online Comments

#### 3.1.1. Web Crawler Technology

Web crawlers download data on Web pages in batches [65], but due to the complex microblog logging mechanism and unified data format, we need to write special crawlers suitable for the microblog. We developed crawlers in Python to simulate microblog login and search, that automatically grabs comments of relevant events on the microblog and extracts relevant information across web pages through crawlers.

#### 3.1.2. Text Mining Method

Original comment texts differ in length and user expression. In addition, original comment texts are mixed with much meaningless content redundant for our analysis. Therefore, we first needed to preprocess the crawled text data using a text mining method. The process is as follows:

① Arrange the text data in chronological order.

② Manually set a user-defined dictionary in combination with popular network words and microblog expressions.

③ Use the Jieba package to set Chinese text segmentation in the text data. Jieba package is one type of tool package in Python, especially for Chinese text word segmentation. The main functions of the Jieba package include text segmentation, user-defined dictionary, keyword extraction and speech tagging. It supports three text segmentation modes: precise mode, full mode and search engine mode [66].

④ Dismiss words based on the stop words list to avoid the interference of irrelevant words.

Emoji in microblog comments often have strong emotions, and they have corresponding Chinese words, which have an impact on the result of word segmentation in the processing. For example, the Emoji 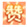, meaning good in Chinese. This can be processed into Chinese and good using Jieba due to its limitations of word segmentation, which affects the calculation of emotional values of sentences. In order to improve the effectiveness of word segmentation, we also added microblog Emoji to the user-defined dictionary. In the process of crawling text data, the words corresponding to the Emoji are obtained through code settings and marked with “[ ]”. Then, based on the use frequency of Emoji and combined with the emotional Dictionary [67], the Emojis with non-obvious emotional tendency are removed. Finally, 59 widely used Emoji were obtained.

In addition, considering the particularity of domestic violence and the popularity of network language, in order to improve the accuracy of test segmentation, this research developed a user-defined dictionary by manual supervision with reference to microblog hot search nouns, popular network words and a microblog expression thesaurus.

The process of constructing a user-defined dictionary was as follows. (1) Obtain microblog topics during the event, mainly extract the most-searched subject words based on name or microblog ID. (2) Select the words to add to the user-defined dictionary based on the occurrence frequency of subject words from the event comment text. (3) Carry out the first segment words and count word frequency. Manually label them according to network catchwords. Extract words with high word frequency or those are easy to be wrongly divided and add them to the emotional dictionary. (4) Summarize and sort out according to the nature of words.

An example of test segmentation for IPV events is shown in Table 2. The test segmentation results with user-defined dictionaries are more accurate than those without user-defined dictionaries (underlined words). For example, in the original sentence, “Domestic violence man” is directly divided into two words through using the Jieba package, namely “Domestic violence“ and “Man”. The word formation is “Verb + noun” or “noun + noun” in the Chinese context. However, with a user-defined dictionary, “Domestic violence man” becomes one word, a noun.

### 3.2. Calculation of Text Sentimental Value Based on Sentiment Dictionary

The sentiment dictionary analysis method mainly judges the sentiment polarity characteristics of the original information corpus through the sentiment dictionary [68]. The construction of the IPV general sentiment dictionary and sentiment domain dictionary will significantly improve the accuracy of sentiment analysis, and these dictionaries need to accommodate a large number of network words and domain words. From a technical perspective, there are three methods to construct a sentiment dictionary: sentiment dictionary construction based on heuristic rules; sentiment dictionary construction based on a graph, and sentiment dictionary construction based on expression learning. This research used heuristic rules to build a sentiment dictionary because it is highly targeted, can extract emotional words in specific fields, and is relatively simple [69]. The construction method mainly reveals grammatical patterns, grammatical rules, semantic features, and language features by observing the characteristics of a large number of corpora. It then extracts emotional words and judges their polarity [67].

#### 3.2.1. Construction of the General Sentiment Dictionary

A general sentiment dictionary or a basic sentiment dictionary refers to a dictionary suitable for most general fields and does not change the sentiment polarity with the change of the professional field. The widely used sentiment dictionaries in the Chinese context are the HowNet emotional database (HowNet Dictionary) and the general Chinese sentiment dictionary of the Taiwan University of China [70]. We selected the HowNet sentiment dictionary as the general dictionary. The HowNet sentiment dictionary covers positive and negative evaluation words and emotional words [67] widely used in mainland China [67,71].

Generally speaking, emotional words change their sentiment polarity after being modified by negative words. We refer to the HowNet negative word dictionary, combine the text features in the IPV event to build a negative word dictionary, and set its weight to −1 [31], as shown in Table 3.

Adverbs of degree are often used to modify or limit adjectives and verbs. Due to the short characteristics of microblog text, degree adverbs are very important for users to express their emotions. The degree adverb dictionary from the HowNet dictionary library is classified into six levels, namely “extremely/most, super, very, relatively, slightly and under”. Give certain weights to these six levels respectively [72,73]; examples are shown in Table 4.

At the same time, the TF-IDF (term frequency-inverse document frequency) algorithm is used to extract the first 100 adverbs to improve the accuracy of text calculation. The TF-IDF algorithm is a classical statistical method to calculate word weight. Its core idea is that the more a word appears in a document, the greater its importance and the more frequently it appears in other documents of the same category. TF means term frequency and indicates the ability of the word to express the content of a document; IDF means inverse document frequency and indicates the ability of the word to distinguish documents. Consequently, the repeated words with the HowNet dictionary database were removed from the first 100 adverbs and, finally, 24 words were retained and listed in the degree adverb sentiment dictionary in the different degree levels.

In addition, 59 emoticon sentiment dictionaries were constructed by capturing emojis that are used frequently. Those that express positive emotions and negative emotions are listed in Table 5.

#### 3.2.2. Construction of a Sentiment Dictionary in the IPV Field

Since the general sentiment dictionary did not include emotional words in all fields, it was necessary to construct a sentiment dictionary to identify the proprietary words in the IPV field.

(1) Use TextRank algorithm to extract seed emotion words.

The TextRank algorithm, its core idea coming from PageRank, obtains the importance of each word by identifying the collinear relationship between words [74]. If a word appears after many different words, it is more important and its TextRank value is higher [75]. In order to improve the accuracy and comprehensiveness of the dictionary, our research used the TextRank algorithm to extract keywords from IPV events in social networks, manually label some emotional words, and label the network vocabulary and popular search words are used as seed emotional words.

(2) Construct the IPV domain dictionary based on the SO-PMI algorithm.

Our research used the SO-PMI algorithm to construct the domain dictionary. PMI is a calculation method proposed by Turney [76] to calculate the similarity between words. The greater the probability of two words appearing simultaneously in the text, the closer the correlation and the higher the degree of correlation. The calculation formula is as follows:

$$\mathrm{PMI}\left(\mathrm{word}1,\mathrm{word}2\right){=\log}_{2}\left(\frac{P\left(\mathrm{word}1\&\mathrm{word}2\right)}{P\left(\mathrm{word}1\right)P\left(\mathrm{word}2\right)}\right)$$

Here, P(word1&word2) represents the probability of word1 and word 2 appearing in the text at the same time, and P(word1) and P(word2) represents the probability of two words appearing independently in the text.

Further, sentiment analysis is integrated into the PMI algorithm to build a representative and domain-adaptive polar dictionary of emotional words [77]. The PMI values of the target words, and the words in the praise vocabulary table and the derogatory vocabulary table, are calculated and subtracted to obtain the sentiment tendency value of the target word. The calculation formula is as follows:

$$\mathrm{SO-PMI}\left(\mathrm{word}\right)=\underset{\mathrm{Pword}\in \mathrm{Pwords}}{\sum }\mathrm{PMI}\left(\mathrm{word},\mathrm{Pword}\right)-\underset{\mathrm{Nword}\notin \mathrm{Nwords}}{\sum }\mathrm{PMI}\left(\mathrm{word},\mathrm{Nword}\right)$$

Here, Pword and Nword represent the words in the positive vocabulary and the negative vocabulary respectively.

SO-PMI(word1)>0 means word1 is the positive word.

SO-PMI(word1)=0 means word1 is the neutral word.

SO-PMI(word1)<0 means word1 is the negative word.

SO-PMI is widely used in the field of sentiment analysis [56]. Using the TextRank algorithm to extract seed emotion words and using SO-PMI to expand the sentiment dictionary of the IPV corpus, can effectively improve the accuracy of subsequent emotional calculations.

### 3.3. Sentiment Analysis

Text sentiment analysis, also known as opinion mining, uses machine learning algorithms to analyze, process, summarize and reason text and can be used to evaluate text sentiment [54,78]. Whether the evaluator’s evaluation or attitude towards IPV events is positive can be analyzed by Text sentiment analysis, and a quantitative analysis of the evaluator’s sentiment can be achieved through the classification of positive, negative, or neutral levels [79].

After the comment text is input, it is analyzed according to the content of the sentiment dictionary to determine the sentiment polarity of the text [80]. The comment text of the IPV event on Weibo can be selected for in-depth analysis to obtain the sentiment orientation and focus of the Chinese public opinion on the IPV event.

Suppose that $v_{i}$ is an emotional word in the review text, ${\mathrm{Av}}_{i}$ represents the weight of ${\;v}_{i}’s$ degree adverb, ${\mathrm{Bv}}_{i}$ represents the weight of $v_{i}$, and ${\mathrm{Cv}}_{i}$ represents the weight of $v_{i}’s$ negative word. The basic algorithms are shown as Algorithm 1.
**Algorithm 1.** Algorithm of sentiment orientation analysis based on sentiment dictionary1.// Calculation of sentiment value2.if $v_{i}$ in sentiment_dict3.  then $E_{i}={\mathrm{Av}}_{i}\times {\mathrm{Bv}}_{i}$
4.if $v_{i}$ in privative_words5.  then $E_{i}={\mathrm{Cv}}_{i}\times {\mathrm{Bv}}_{i}$
6.$E_{\mathrm{si}}=\overset{k}{\underset{i=1}{\sum }}E_{i}$ // the sentiment tendency of each sentence scores7.$E=\overset{n}{\underset{i=1}{\sum }}E_{\mathrm{si}}$ // total score of sentiment tendency of single comment text on Weibo8.// Analysis of sentiment tendency. The rules of classification are as follows.9.if E > 010.  then E is positive11.  else if E = 012.     then E is neutral13.     else E is negative

The process is as follows. First, perform part-of-speech tagging of emotional words, traverse the sentiment dictionary, negative word dictionary, and degree adverb dictionary, and perform emotional calculation on the processed text. Second, calculate the emotional value of each word in the sentence based on different weights, and obtain the emotional value of the sentence through accumulating the word value. Finally, classify positive, neutral, and negative emotions based on the emotional value of sentence. Sentences whose values are greater than 0 reflect positive emotions, those less than 0 express negative emotions, and those equal to 0 express neutral emotions.

## 4. Analysis of Public Attention and Sentiment

### 4.1. Data Acquisition and Data Process

#### 4.1.1. Data Acquisition

The home page of *Yuya* on Weibo contains four blogs. Two blogs were published on 25 November 2019 (to disclose domestic violence on the Internet), the third blog on 28 November (case handling results of the police) and the last one on 5 December (response to netizens in terms of her physical and mental conditions). Meanwhile, official accounts of China’s official media were actively forwarding blog posts to attract the attention of many netizens. *People’s Daily* updated the progress of the Chongqing police in handling the case; *The Paper* (a famous news media publication in China) updated the results of the case and encouraged the victim to protect her rights and interests bravely.

By crawling comment texts of the four Weibo contents and relevant posts released by official accounts on social media (such as *People’s Daily*, *The Paper* and *NewsHead*) through the Python crawler software, 34,350 comment data were obtained, including comment time, nickname, ID and comment text. In order to obtain more attributes of comment data to analyze different characteristics of people’s attitudes towards IPV in depth, the information of Weibo users discussing this event was also crawled to obtain attribute information such as the location, gender and age of users.

#### 4.1.2. Data Preprocessing and Descriptive Statistics

**Figure 2:**
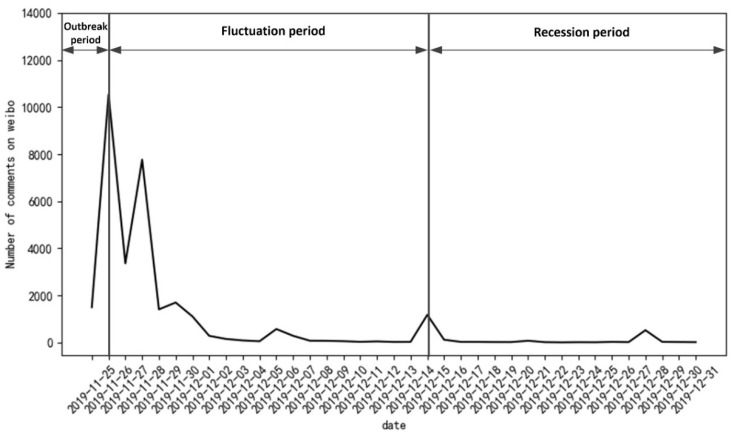
Trends in the comment intensity of the *Yuya* event.

First, because some comments involve advertisements or have no specific meaning, it was necessary to delete repeated, missed, invalid and irrelevant data in the comment dataset. After preprocessing, 31,323 comment data remained. Comments on Weibo were sorted by the number of “likes” by default. Figure 2 shows the comment popularity of *Yuya* event in the time series. Considering that the fluctuation amplitude of the number of comments tended to be gentle after 28 December 2019, the life cycle of the event of *Yuya* on Weibo was from 25 November to 31 December 2019; the amount of comment data peaked on 26 November 2019.

#### 4.1.3. User-Defined Dictionaries in IPV

Considering the specific Internet environment in the Weibo and IPV fields, this study set up four types of user-defined dictionaries by combining Internet buzzwords and the Weibo emoticon database, as shown in Table 6. Based on the user-defined dictionaries, the Jieba package in Python was used for Chinese word segmentation, and the stopword list developed by the Machine Intelligence Laboratory of Sichuan University was selected to stop words.

#### 4.1.4. Sentiment Analysis

According to the previous method, the emotional values of comments are calculated and the emotional tendency is judged. The specific process is shown in Section 3.3.

**Figure 3:**
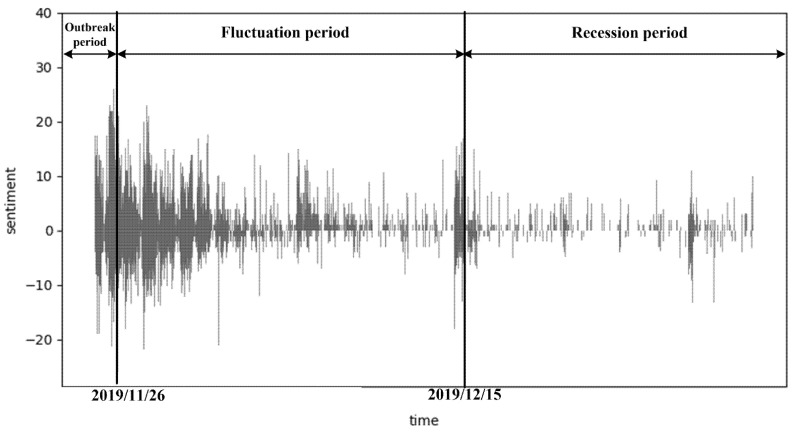
Trends in the evolution of public sentiment of the *Yuya* event.

As displayed in Figure 3, the sentiment analysis of the comment data from 25 November to 31 December 2019, reflects the emotional states and development process of the public.

### 4.2. Analysis of Time Series

By referring to the theory of public sentiment [67], the development stage of hot events on the Internet is generally divided into an initial stage, an outbreak stage, a development stage and a recession stage. The blogger of *Yuya* event chose to release the video in the afternoon of the International Day for the Elimination of Violence against Women. Under the dual effect of internet celebrity and the topic of “anti-domestic violence”, the entry of “*Yuya* suffering from domestic violence” quickly entered the outbreak period and topped the hot topics of Weibo on that night.

**Figure 4:**
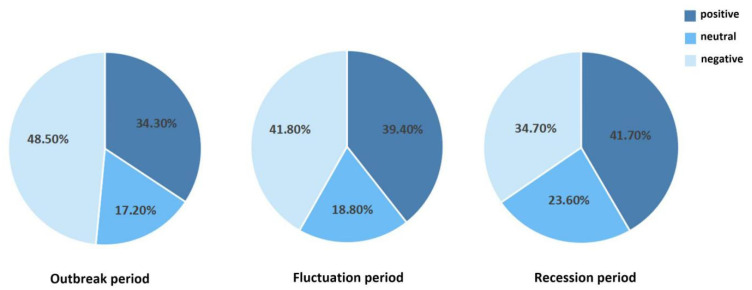
Sentiment classification of the *Yuya* event.

**Figure 5:**
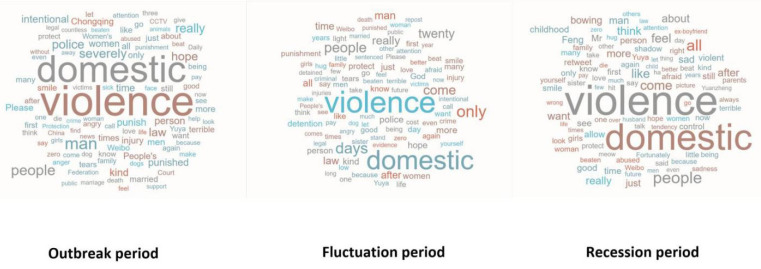
Subject headings clustering of the *Yuya* event.

Based on important time nodes, number of comments, and concentration of negative emotions, the event of *Yuya* was divided into three stages (T = 3), (I) outbreak stage, (II) fluctuation stage and (III) recession stage, as demonstrated in Figure 3. Furthermore, Figure 4 displays the statistical description of public emotional tendency in the above three stages. The subject word frequency of the comment text in the three stages is illustrated in Figure 5, using cluster analysis.

Stage 1: Outbreak period

Stage 1 started on 25 November and ended on 26 November 2019, in which negative, neutral and positive emotions of the public accounted for 48.5%, 17.2% and 34.3%, respectively. The intensity (or value) of negative emotions of the public was concentrated between −10 and 0, and the smallest value was −26, which is lower than the overall value of negative emotions. Positive emotional intensity (or value) was concentrated in 0~10, with the largest value at 26. The absolute value of the sum of all negative emotions was generally larger than that of positive emotions. This indicates that at this stage, the prevailing emotional attitude of the public was rational thinking.

As shown in Figure 5, in addition to subject keywords (such as “domestic violence”, “hope” and “protection”) commonly shown in the three stages, those like “severe punishment”, “intentional injury”, “call the police”, “male perpetrator of domestic violence”, “*Women’s Federation*” and “*CCTV*” were highly frequently used in the outbreak period. Particularly, the word “anger” reflects the extreme emotion of the public. The public generally resents and dissatisfies with the IPV event and male perpetrator of domestic violence and expresses their attitude that the man must be severely punished, even including extreme views, such as “death penalty”, “castration”, “down to hell” and “shooting”.

Keywords including “call the police”, “*Women’s Federation*” and “law” suggest that some members of the public have a deep understanding of the nature and handling mode of the IPV event and put forward reasonable suggestions.

Stage 2: Fluctuation period

The duration of this stage was from 27 November to 15 December 2019. The intensity (or value) of negative emotions was between –5 and 0, and the lowest value was –21.75; the intensity (or value) of positive emotions was still concentrated between 0 and 10, with the highest value at 23. The absolute value of positive emotions was generally higher than that of negative emotions. This indicates that the emotional attitude of the public changed, and rationality was gradually gaining ascendancy in this stage.

During the fluctuation period, there were many subject keywords in Weibo comments, such as “Legal Report”, “twenty days”, “call the police,” and “retaliation”. In particular, “come on” and “hope” reflect positive emotions of the public to the event.

*People’s Daily* published that #the man suspected of domestic violence against blogger *Yuya* has been arrested# at 8:00 a.m. on 28 November. Then, at 9:00 a.m. on 28 November, the Weibo account of Chongqing Public Security Bureau (@*Ping’anChongqing*) released the case processing results. On 15 December, “Legal Report” on *CCTV* broadcast a special interview program of *Yuya*, which made the domestic violence event public again. The interview and report of “Legal Report” informed more people of the cause and course of the event. However, compared with the outbreak period, the negative tendency decreased. The focus of public attention gradually shifted from the perpetrator of domestic violence to encouraging and supporting the victim. This shows that the authoritative information released by China’s official media played a vital role in calming public sentiment.

Stage 3: Recession period

This stage lasted from 16 December to 31 December 2019. In this stage, the number of social network users discussing the event of *Yuya* decreased significantly. As the public opinions on this IPV event gradually lessened, the number of positive, neutral and negative emotions in the comment text were relatively even.

Generally speaking, there was increasingly less news about the event on Weibo in the recession stage. The netizens encouraged *Yuya* with positive emotions, but their attention was gradually diverted by other events.

On 28 December, the topic of “how to identify a partner with domestic violence tendency as early as possible” was published on *China News Service*, with pictures of *Yuanzheng Feng* (an actor who once played a role of a man committing domestic violence). Moreover, common characteristics of people with domestic violence tendencies were proposed, such as poor emotional control, the strong desire for control and strong suspicions. *Yuanzheng Feng* created the image of men committing domestic violence in TV dramas. In Figure 5, words such as “childhood trauma”, “*Feng Yuanzheng*”, “control”, “politely suppressing laughing and ridicule when others share some embarrassing experience” (Weibo emoticon) and “sadness” (Weibo emoticon) reflect negative emotions of people. The mainstream media guided the public to understand IPV more widely through agenda-setting.

The anonymity and interactivity of the Internet make people break through the moral restraints and supervision of public social opinions in society to express their real emotions truthfully or more forcefully. It is worth mentioning that swearing and extreme words were particularly common during the outbreak period of the event, considered as the venting of negative emotions. In the online world, although there are some defects in communication with people through words compared with sound and vision, these defects do not hinder users from communicating and can express more emotional information. It is difficult, in real life, to determine emotional characteristics on the Internet. Therefore, we need opinions expressed on the Internet to understand the views and attitudes of the public towards IPV truly.

### 4.3. Analysis of Geographical Space

The location of users enriches social media data and provides multiple-perspective analysis for the research [81]. Authenticated users on Weibo are distributed globally: domestic users account for 72.55%, overseas users account for 6.78% and 20.67% of users who do not fill in regional information.

In general, the higher the level of regional economic development, the higher the social status of women and children, and the higher attention from Women’s Federations and grass-roots communities to IPV events [36]. By referring to the division of four major economic regions in China [78], the regional distribution of the public concerning the *Yuya* event was shown as follows: eastern (55.5%), western (23.3%), central (15.4%) and northeast regions (5.9%) are ranked in descending order.

**Figure 6:**
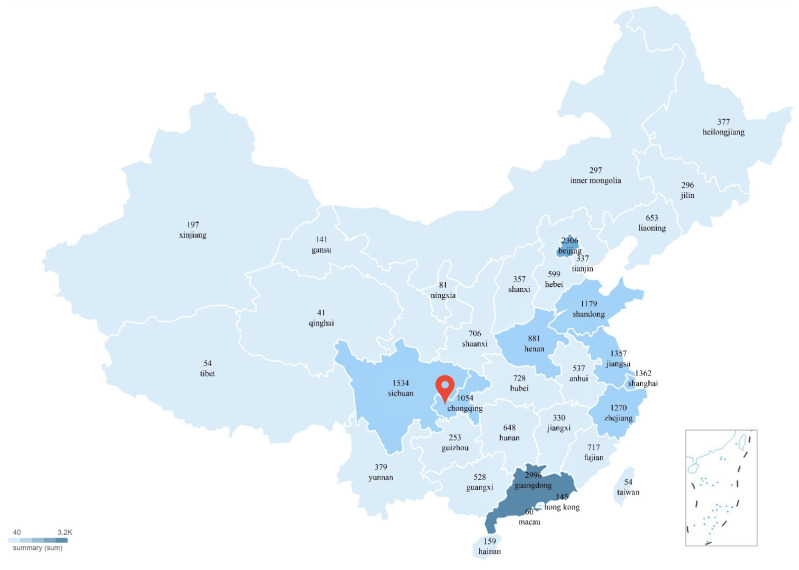
The spatial distribution of public sentiment of the *Yuya* event.

To be specific, the public in Beijing, the Pearl River Delta and the Yangtze River Delta paid close attention to the IPV event, as shown in Figure 6. These regions have a high degree of economic development and are the richest regions in China. Therefore, these regions had higher concerns about the IPV event, which supports the study of Song et al. (2021).

The public in Shandong and Henan were also actively concerned about the *Yuya* event. According to Wu Jiezhen’s report [82], Shandong and Henan provinces are the regions with the highest number of suspected cases of domestic violence, with 8205 and 6986 respectively. In November 2018, the Standing Committee of Shandong Provincial People’s Congress passed the first regional regulation concerning antidomestic violence in China. This directly indicates that the degree of concern about IPV events is related to domestic violence events, and also shows that the legislation and law popularization of the government can effectively increase the public’s attention to IPV events.

Meanwhile, the public in Sichuan Province paid close attention to the *Yuya* event because the case occurred in Chongqing (Chongqing once belonged to Sichuan Province and became a municipality directly under the central government since 1997). In the comment text, on the one hand, Weibo users in Sichuan province expressed their anger at the perpetrator; on the other hand, they were ashamed because they were in the same geographic location as the perpetrator, this being especially so for people in Chongqing City.

In underdeveloped regions where the education and health of women are at a low level, many women have experienced IPV, but they have not enough resources to protect their rights and interests, which is also reflected by lower public attention in underdeveloped western regions.

### 4.4. Guiding Role of Social Media on Public Sentiment

In this IPV event, government agencies, China’s official media, mainstream media and social media platforms played a particularly important role in guiding opinions concerning public cognition, value, attitude and behavior. In this IPV event, government agencies represented by the official microblog of the Chongqing police swiftly released the results of the IPV event. China’s official media, represented by the *People’s Daily*, and mainstream media, represented by *The Paper* and Legal Report, as opinion leaders guided public opinions through agenda-setting. The social media platforms represented by *Today’s Headlines* made personalized recommendations based on personal interests. This addressed the problem that the celebrity effect of the victims in the IPV events (mainly violence against women) obscures other attributes of IPV (violence against men, same-gender violence, sexual violence, economic violence, etc.), and enlarged the discussion scope of IPV events. Meanwhile, this also played a key role in promoting the positive change of public attitudes and emotions and the rapid entry of public opinions on the Internet in the recession stage.

The police play important roles as law enforcers, mediators, and social workers in the prevention and control of domestic violence and are powerful voices in the social network environment. The District Branch of Chongqing Public Security Bureau cooperates with Chongqing Women’s Federation to communicate directly with the public through social media, such as government microblogs, and responds to public concerns swiftly when IPV events occur, so it plays a vital role in guiding public opinions. The police and Women’s Federation have promoted the positive development of attitudes and emotions of the public and have carried out legal publicity of IPV events for the public by setting a public agenda.

Generally, through agenda setting, mainstream media can determine the nature, and make attribution and moral evaluation of IPV events, and put forward solutions, which can enhance the public’s understanding of IPV events and effectively guide public opinions on the internet.

Usually, hot events mostly follow the propagation path of launching the agenda by social media, then follow-up by mainstream media and agenda diffusion. However, in the *Yuya* event, the media agenda was initially set by the victim herself on social media; called reverse agenda-setting by some scholars [83]. Secondly, the government media agenda and mainstream media agenda played their respective roles in different stages of public opinion and realized agenda convergence, which is the process of agenda integration. Once the *Yuya* event occurred, it quickly generated online public sentiment, and the mainstream media followed its progress at all times and forwarded the blog released by the police on the topic of #police investigation of domestic violence suffered by *Yuya*#. Besides this, mainstream media also focused on how to identify a man prone to domestic violence and issued questionnaires to explore what measures the public may take to address domestic violence, and to guide the public in understanding and treating domestic violence correctly. Although the mainstream media did not intervene first in this event, they played an important role in stabilizing public sentiment and expanding topics during the development and recession stages of public opinion. In the long run, this will affect the action orientation of people against domestic violence.

## 5. Further Discussion

Blake et al. [84] proposed that domestic and family-violence events are positively correlated with the number of female misogynistic tweets in these areas. On this basis, we mined the microblog data thoroughly and found that misogyny was reflected in verbal, emotional, sexual, economic and psychological abuse as an expression of extreme negative emotions in IPV events. In fact, the anonymity of the social network enables people to express their innermost opinion more freely, and the guiding role of opinion leaders results in a spiral of the anti-silence effect.

### 5.1. Misogynic Characteristics in the IPV Event

Misogyny and domestic violence are global phenomena [84]. Misogyny not only occurs in face-to-face interactions in real life, but also in the form of publishing discriminatory words about women on social media. Misogyny is usually embodied through disrespectful, hostile and sexist emotions and attitudes, is shown in sexual objectification of women, endorsement of male dominance over women, and endorsement of rape culture [85]. It is manifested as man’s “contempt for females” and women’s “self-loathing”. There is no research to explore the phenomenon of misogyny in China. Affected by the traditional culture of male chauvinism and the modern male gaze, the vast majority of Chinese people have not been aware of the existence of misogyny or have been ashamed to agree with the idea of misogyny.

With mobile internet development, people can express their thoughts, emotions, and feelings through social media regardless of time and place, and social media has become a mainstream channel of communication [86]. It is easier to express misogynistic attitudes and malicious emotions through social media than through face-to-face interaction. The #MeToo and #TimesUp campaigns highlight how individuals are using new technologies to connect with other survivors of sexual assault and share their stories on social media [87]; as a result, social media provides us with more access to content that discriminates against women. Due to the potential delicacy of the Chinese language, it is difficult to identify misogynistic content automatically. Therefore, this study manually identified texts with misogynistic characteristics in the comment data of the *Yuya* event, such as slander, domination, sexual harassment, stereotype and infidelity.

The specific characteristics of misogyny are reflected in verbal, emotional, sexual, economic and psychological abuse. The perpetrator’s violence appears to be an emotional catharsis but, in fact, it contains hatred against women at its heart. Verbal abuse of the victim is the actual embodiment of misogyny. More importantly, the comment texts in Table 7 show that in the context of a male-dominated society, individual assumptions concerning the reason why female IPV victims do not leave the offender is because of resources (power and money) and the sexual characteristics of men.

Negative attitudes towards women also increase the risk of relationship violence. After domestic violence occurs, the actual violence may appear on the internet [84], and comments reflect the fact that few people do not welcome women. Furthermore, the attitude of discriminating against women on the internet may incite some men to carry out domestic violence in real life. Therefore, the only way to combat online violence against women is to challenge gender inequality in the wider society. This requires relevant departments, such as governments, to continue their efforts to carry out scientific popularization of IPV events and guide the public to adopt correct values.

### 5.2. The Spiral of Silence Effect in the IPV Event

In Chinese traditional family culture, IPV is regarded as a personal matter and should be handled within the family, so Chinese women are unlikely to disclose IPV events [10]. Moreover, due to “face-saving”, some victims believe that telling their experience of suffering from domestic violence will produce a sense of shame, so they choose to tolerate IPV and blindly believe that the situation will change. In this way, they cannot obtain external intervention and rescue in time and may even suffer from more frequent domestic violence. Previous studies [10,88,89] have demonstrated that the public often avoids talking about domestic violence topics because of “face-saving”.

Social media’s high freedom and openness enable the public to break through the boundaries of time and space to understand IPV events and express their personal views directly and deeply. Particularly, when celebrities suffer from IPV, this is accompanied by entertainment, triggering public opinions and topics on the internet. Netizens actively express their personal emotions of anger and sympathy and even comment on their own experience. After an internet celebrity, *Papi* supported *Yuya* in public, a positive response was received from netizens. This indicates that authoritative media and Internet celebrities (microblog users who have obtained real name authentication on microblog platforms, such as Sina, Tencent and Netease and have many fans) participating in the discussion have strong discourse powers and influence as opinion leaders with a huge fan base.

On the one hand, the spiral of silence theory, which was once popular in mass communication, has undergone some changes in the era of social media. When people judge that their opinions are different from those of the majority, they no longer remain silent, and the guidance of opinion leaders allows the public to more actively express their views and attitudes. When the event that “stars suffer from domestic violence” was published on the social media platform, the public was more participatory and could freely express or support the opinion of the “minority” to encourage victims to speak and give them sympathy and assistance. More netizens further accept the opinion of the “minority” and the traditional opinion of the “majority” (that family shame should not be publicized) is being gradually changed under the fast spread of public opinions, which accepts and supports the courage of victims to speak.

The essential attribute of the spiral of silence theory is not short-term opinion expression but medium and long-term media effects. Throughout the outbreak, fluctuation and recession cycle of the *Yuya* event on the internet, public behaviors, including public opinion expression of the “minority”, guidance of opinion leaders, insistence of backbone supporters and active participation of internet users, developed strongly in the anonymous, virtual and interactive environment of social networks. This resulted in a spiral of anti-silence. The public’s approval for supporting victims is conducive to the protection of victims’ rights but also provides an example for potential victims.

On the other hand, the spiral of silence theory still has its theoretical applicability in the emotional communication process of IPV events. In the *Yuya* event, the majority of comments expressed extreme emotions, especially questioning the light punishment given by the police. They had more “likes”, and quickly became the “majority” after the punishment of administrative detention for 20 days for the perpetrator was released. However, users’ comments expressing a belief that the punishment was relatively scientific and objective became the “minority” and were swamped by the comments of the “majority”. These results show that the public with more extreme emotions is more likely to become the “majority”, and more neutral and rational emotions are likely to be “drowned”. At the same time, due to group pressure and conformity psychology, individuals are willing to agree with the opinions of the “majority”. Hence, the spiral of silence theory arises, and the phenomenon of violence on the internet against perpetrators also occurs. In particular, the popular comment mechanism of Weibo cannot only allow Weibo users to intuitively feel public emotions, but also promote the spread of extreme emotions and the intensification of the spiral of silence to a certain extent.

In conclusion, the anonymity of posts on social media and the guiding role of opinion leaders can result in traditional cultural factors, such as “face-saving” and “family shame” for IPV events to be no longer applicable, allowing the public to be no longer silent. In the spreading of public emotions, anger often overwhelms rationality, and the spiral of silence effect still plays an important role.

## 6. Experts Opinion Based on the Delphi Method

### 6.1. Participants, Design, and Procedures

To supplement our research findings we used the Delphi method to test the completeness and reliability of the discussions in this study. The Delphi method allows experts to work toward a mutual agreement by conducting a circulating series of questionnaires and releasing related feedback to further the discussion with each subsequent round. This method seeks to aggregate opinions from a diverse set of experts, and it can be done without having to bring every expert together for a physical meeting. Since the responses of the participants are anonymous, individual panelists do not have to worry about repercussions concerning their opinions.

According to the expert selection principle of participants being professional, authoritative and comprehensive, an expert in domestic violence (a psychological counselor who has worked for more than ten years), a data science professor (a professor of big data science at a university), a Women’s Federation leader (with related work experience for more than five years), a public health official (deputy director-level cadre of the health department), and a microblog opinion leader (over one hundred thousand followers on Weibo) were selected as survey experts. These experts were chosen with the knowledge that we were researching domestic violence.

A survey questionnaire with five questions was created, and respondents were asked to provide their opinions on whether misogyny and the spiral of anti-silence effect has been broken in the context of the internet.

The Delphi method in our study involved two rounds. First, each expert was sent a questionnaire with instructions to comment on the topic based on their personal opinions, experience, or previous research. Five questionnaires were distributed by email, and five questionnaires were returned, with an effective rate of 100%. In the second round of discussions, we provided the five participants with the aggregated results of the first round of the survey and an indicator importance score table. Evaluation indicators included the generality and applicability of the research framework, the spatiotemporal characteristics of IPV, the role of mainstream media and opinion leaders, the misogyny characteristics in IPV events, and the spiral of silence effect in IPV events. The rating scale was measured using a 5-point Likert scale (i.e., “How important is this issue to you?”; 1 = “not at all”; 5 = “very much”).

### 6.2. Results and Discussion

In general, all experts agreed that domestic violence has moved from the private sphere into the internet environment, attracting attention and discussion of netizens all over the country, which represents a great contrast with the past and has overturned the spiral of silence theory. In addition, all participants agreed that opinion leaders and mainstream media play an essential role in public opinion control. Traditional ideas and victim-blaming theory affect some people’s judgment of domestic violence cases and result in a new discourse of violence concerning the victimized women.

Next, the results indicated that the indicator of misogyny characteristics had stronger significance in IPV events. Further, we asked the participants whether they thought that victim-blaming was a common discourse strategy behind the misogyny phenomenon in social media. If it is recognized, why do people blame the victim? All participants believed that this view was rooted in the basic cognition of women and gender relations in Chinese traditional culture. The potential subconscious of “it is by no means accidental” behind so-called “family conflict” must influence people’s judgment of gender violence cases. Statements, for example, “flies don’t bite seamless eggs” are usually inflammatory in the network environment. To some degree, the open platform, network evaluation system and communication forms dispel the awareness of the traditional “disgusting women” and form a new discourse strategy.

The experts believed that big data provides new insights and conclusions for our research, unlike traditional research. Computational methods can be used for studying domestic violence and are effective. The leader of the Women’s Federation believed that this research shows the problems existing in reality from a novel perspective, and we still have much work to do. The public health official believes that such research will help people gain a deeper understanding of China’s public health problems and the attitudes of the Chinese public, thereby facilitating relevant policy formulation. The opinion leader said that this research has played a crucial role in developing public opinion and is very meaningful.

Finally, we used Kendall’s coefficient of consistency (Kendall’s W) to test the robustness of the pooled results of expert opinions. As a nonparametric statistical method, Kendall’s W can be used to measure the consistency of multiple sets of rank values. The value range of Kendall’s W is from 0 to 1. The larger the coefficient, the stronger the consistency of multiple sets of data. Kendall’ W was greater than 0.6 with statistical significance at *p* < 0.05, indicating that the five participants reached a consensus during the Delphi process.

These findings prove that the data and methodology are proper for this study. These findings support our conclusion that misogyny culture is reflected in domestic violence cases. The anonymity of the internet and the guidance of opinion leaders promote the spiral of the anti-silence effect.

## 7. Conclusions

Using the methods of text mining and emotion analysis, this research used the *Yuya* event as an example to analyze evolution of the Chinese public’s sentiment concerning IPV from the perspectives of a time series, geographic space and social media.

First, the public in China has become concerned about IPV events and encourages victims to seek legal help. The finding shows that netizens in economically developed areas pay close attention to IPV events. The vast majority of netizens hate perpetrators and have the attitude that perpetrators should be punished. Negative emotions ran through the entire course of this IPV event. Furthermore, the public believes that IPV is illegal and supports punishing perpetrators through legal means. People provided affirmation and encouragement to the victims’ disclosure of domestic violence, which expands the cognitive scope of IPV.

Secondly, China’s official media, mainstream media and opinion leaders play a particularly positive role in guiding public opinions from negative emotions to rational analysis. They set the media agenda to enhance the public’s understanding of IPV events. In the long run, this will affect the action orientation of people against domestic violence.

Thirdly, the spiral of silence theory in the era of mass communication has been challenged in the era of social media. Influenced by traditional ideas, the public in China generally believes that domestic violence occurs within the family, and family shame should not be publicized. In this event, the Chinese public began to disclose and discuss IPV, indicating the public believes that IPV events are no longer private family affairs. However, this does not mean that the theory is no longer applicable. In the process of public emotional communication, anger often overwhelms rational analysis, resulting in silence. The spiral of silence effect still works well in the era of social media.

Finally, a few offensive words accompanied by comment texts on IPV show misogynic characteristics, regardless of the victim being a man or woman. This abuse is often reflected in emotional, sexual, economic and psychological abuse. However, under the influence of traditional culture, victim blaming is a common discourse strategy behind the phenomenon of misogyny, which affects people’s judgment of gender violence events. In addition, in social media, the network evaluation system and the form of communication dispel the emotions of misogyny to some extent and form a new discourse strategy.

### 7.1. Theoretical Implication

First, we applied the methods of text mining and sentiment analysis to study the IPV event by collecting data from social media. This improves the previous literature that adopted interviews or questionnaires to survey. This method can be used in the field of IPV and other public health research, and shows that social media data is very useful for research in the field of public health and can analyze public health problems from a new perspective.

Second, this study expands research in the field of misogyny culture and further explores the embodiment of misogyny culture in domestic violence. Misogyny is a new discourse strategy in the social media environment, is a common phenomenon in real life and also occurs in social media through text expression. Through text analysis, this study found that victim-blaming in domestic violence is a common discourse strategy behind misogyny, which supports the study of Blake et al. [84].

Third, our research explored the applicability of the spiral of silence theory in the social media environment. Although relevant studies show that the spiral of silence theory is still applicable in the online social environment, willingness to self-censorship predicts that individuals keep their opinions to themselves [90]. Our study found that the public no longer silent in the network environment but actively discusses IPV, resulting in a spiral of anti-silence. This study makes up for the lack of such research and expands research on the spiral of silence theory.

### 7.2. Policy Implications

The problem of domestic violence has gradually transitioned from the private sphere to the public sphere, and has become a social problem worthy of profound reflection. The Chinese government should be highly attentive to the problem of domestic violence.

The credibility of a government is essential. Having credibility means that government departments can be trusted and recognized by the public when publishing the outcome of cases. At the same time, it is necessary to improve the construction of laws and regulations against domestic violence and improve the publicity of law promulgation conducive to the formation of legal awareness of antidomestic violence, and safeguarding rights throughout the entire society. In addition, the government needs to pay attention to the whole course of events in particular situations and take timely measures to inform public opinions resulting from emergencies.

In fact, as a new supervision tool, the microblog is a public opinion forum for internet users to independently exercise supervisory power to a certain extent. Mainstream media can play a positive guiding role for network opinion leaders by public opinion supervision. Combining public opinion supervision with positive publicity, the mainstream media can improve the ability of people to judge the validity of network information and strengthen users’ self-discipline and rational expression by interpreting national publicity guidelines, policies and laws.

### 7.3. Limitations and Further Study

This study analyzes the evolution of public attitudes and emotions from online comments data. The case selected in this study was specific, and the rapid spread of information concerning the event was inseparable from the identity of the internet celebrity victim. People naturally sympathize with victims. However, when a sexual minority (lesbians, gays, bisexuals, transgender, and queer) choose to seek help on social networks after experiencing IPV, it is still unknown whether they attract public attention and are supported by public opinions. Therefore, it is necessary to further explore the cognitive scope of Chinese people concerning IPV, and study how to prevent and safeguard the rights and interests of victims. In a future study, we will further address the burning issue of domestic violence, improve the IPV emotion dictionary and improve its applicability.

## Figures and Tables

**Table 1:** Summary of methodology in the field of IPV.

Author (Year)	Measure	Sample Size	Conclusions
Lin et al. [33]	Questionnaire	491	Gender-role attitudes, such as beliefs of male dominance and IPV as crime, were among the most prominent predictors of students definitions of IPV.
Reuter et al. [16]	Questionnaire	172	Studies examining the impact of IPV on negative outcomes and revictimization over time may guide our understanding of the immediate and delayed consequences of IPV for LGBT young people.
Wei et al. [47]	Questionnaire	431	This study quantified the experience of IPV among MSM in China and explored some factors associated with IPV experiences such as self-esteem and the age of first homosexual intercourse.
Pugh et al. [45]	Questionnaire	1153	Viewing IPV as a crime, gender, and beliefs of the causes of IPV were robust predictors of college students’ perceptions concerning why women stay in physically abusive relationships.
Žukauskienė et al. [24]	Interviews	1173	Exposure to different types of IPV was found to be associated with age, relationship status, household income, area of residency, and violence experienced in childhood.
Storer et al. [48]	Thematic content analysis	3086	Seven primary themes emerged that influenced their decision to stay processes: (a) impact of IPV on personal well-being, (b) not identifying as a stereotypical IPV victim, (c) fear of reinforcing racial stereotypes.
Alvarez-Hernandez et al. [49]	Content analysis	29	Eight manifest messages related to seeking help when experiencing IPV in times of a crisis: (1) contact a professional resource, (2) contact law enforcement, (3) contact family, friends, and members of your community.

**Table 2:** Example of preprocessing Weibo comments of IPV.

Original sentence	很遗憾是因为这条微博才认识的美妆博主宇芽一定要加油呀远离家暴男希望你健康快乐 It’s a shame because this microblog is known to beauty blogger Yuya must refuel ah away from domestic violence men hope you are healthy and happy
Test segmentation without user-defined dictionary	很 遗憾 是因为 这条 微博才 认识 的 美妆博主宇芽 一定 要 加油 呀 远离 家暴 男 希望 你 健康 快乐 It’s, a shame, because, this, microblog is, known, to, beauty blogger Yuya, must, refuel, ah, away from, domestic violence, men, hope, you are, healthy, and happy
Test segmentation with user-defined dictionary	很 遗憾 是 因为 这 条 微博 才 认识 的 美妆 博主 宇芽 一定 要 加油 呀 远离 家暴男 希望 你 健康 快乐 It’s, a shame, because, this, microblog, is, known, to, beauty, blogger, Yuya, must, refuel, ah, away from, domestic violence men, hope, you are, healthy, and happy
Stop word removal	遗憾 微博 认识 美妆 博主 宇芽 加油 远离 家暴男 希望 健康 快乐 a shame, microblog, known, beauty, blogger, Yuya, refuel, away from, domestic violence men, hope, healthy, and happy

**Table 3:** Negative word dictionary.

Weight	Negative Word	Quantity
−1	不(no) 甭(don’t need) 勿(shouldn’t) 别(stop) 未(not yet) 反(contrary) 没(without) 否(not) 木有(don’t have) 非(non) 无(none)	70

**Table 4:** General sentiment dictionary.

	Level	Weight	Quantity
Basic sentiment dictionary	Positive, negative	1, −1	8848
Degree adverb	extremely/most, super, very, relatively, slightly and under	2, 1.5, 1.25, 1.2, 0.8, 0.5	243
Emoji	Positive, negative	1, −1	59
User-defined sentiment dictionary	Positive, negative	1, −1	175
SO-PMI	Positive, negative	1, −1	5270

**Table 5:** Positive emoticon and negative emoticon in Weibo.

Positive Emoticon		Negative Emoticon	
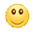	smile	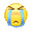	tears
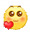	love you	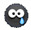	bad luck
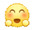	too happy	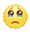	sadness

**Table 6:** User-defined dictionaries for the IPV event in Weibo.

Type	Dictionary
Event-related subject	*Yuya*, *Jiang Jinfu*, *Bancangsenlin* (Weibo user *Wenjie Hu*), *Chen Hong*, *Liu Yang*, *Master Feng* (actor *Yuanzheng Feng*), *Lantai* (Zhejiang Satellite TV Channel, dubbed because of its blue logo), *Yixiang Gao*, *Jianguo Chuan* (*Donald John Trump*, dubbed by Chinese netzines), Sanse Kindergarten (red, yellow and blue)
Official vocabulary	Law on Family Violence, public security organization, *People’s Daily*, *CCTV*, *Yangma* (the People’s Bank of China), *Ping’an Yuzhong* (official microblog account of Yuzhong District Branch of Chongqing Public Security Bureau), *China Woman’s News*, *Chinese Red Cross Foundation*, *Weibo law*, *Central Committee of the Communist Youth League*, *Photography on Weibo*, *China Police Network*, *China Small Animal Protection Association*
Event-related vocabulary	imprisonment, idiot, fuck, difficult to get along with, male, female, okay, damn it, schoolgirl, next one, playboy, scumbag, older adult, male perpetrator of domestic violence, zero tolerance, love, history of domestic violence, history of cheating, more, everything goes well, faithless, plain-speaking, free, shy, dregs like a dog, implantation of contraceptive devices, a writ of habeas corpus, make trouble, excessive, perpetrator of domestic violence, phoenix man (hardworking man from a poor background, generally used to describe those who are sensitive, self-abased, and arrogant)
Weibo emoticon	love you 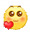 , sad 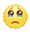 , Chinese praise 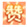 , puzzling 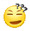 , nosepick 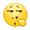

**Table 7:** Texts with misogynistic characteristics in IPV events.

Time	Gender	Comments
11/26 00:00:04	Female	*He does not beat you every day. Do your parents give your hands for masturbation for him? You will endure it again and again, yes? Does he fuck you well? Ho ho, I really do not understand it.*
11/25 22:02:09	Female	*Why not break up quickly after being beaten for the first time? There must be some causes for the abnormal behaviors.*
11/25 22:23:59	Male	*This man must be rich.*
11/26 12:44:24	Female	*For such figure and appearance, I do not believe that the woman is not pursuing for money.*
11/27 23:21:18	Male	*This woman is a bitch. Isn’t it because the man has strong sexual ability?*
11/27 09:34:01	Male	*Isn’t it because you love money? Shouldn’t you deserve it? There must be a reason, otherwise, who will beat you for no reason.*
11/25 22:43:59	Female	*It is you that find the man among scumbags, so you should not only cry about being beaten, but also share the pleasure he gives you. Just reap the consequences. You should not blame anyone.*
11/27 12:04:52	Male	*He should be your old, rich boyfriend. I guess one is for money and the other is for sex.*

## Data Availability

The data presented in this study are available on request from the corresponding author.
